# Safety and effectiveness of non-vitamin K oral anticoagulants versus warfarin in real-world patients with non-valvular atrial fibrillation: a retrospective analysis of contemporary Japanese administrative claims data

**DOI:** 10.1136/openhrt-2019-001232

**Published:** 2020-04-01

**Authors:** Shun Kohsaka, Jun Katada, Kumiko Saito, Aaron Jenkins, Benjamin Li, Jack Mardekian, Yasuo Terayama

**Affiliations:** 1 Department of Cardiology, Keio University School of Medicine, Tokyo, Japan; 2 Internal Medicine Medical Affairs, Pfizer Japan Inc, Tokyo, Japan; 3 Cardiovascular Medical Department, Bristol-Myers Squibb K.K, Tokyo, Japan; 4 Department of Patient & Health Impact, Pfizer Inc, New York, New York, USA; 5 Global Biometrics & Data Management, Pfizer Inc, New York, New York, USA; 6 Neurological Institute, Shonan Keiiku Hospital, Kanagawa, Japan

**Keywords:** warfarin, direct oral anticoagulant, NVAF, stroke, bleeding

## Abstract

**Objective:**

To assess the safety (ie, risk of bleeding) and effectiveness (ie, risk of stroke/systemic embolism (SE)) separately for four non-vitamin K oral anticoagulants (NOACs; apixaban, dabigatran, edoxaban and rivaroxaban) versus warfarin in Japanese patients with non-valvular atrial fibrillation (NVAF), including those at high risk of bleeding and treated with reduced doses of NOACs.

**Methods:**

We conducted a retrospective analysis of electronic health records and claims data from 372 acute care hospitals in Japan for patients with NVAF newly initiated on NOACs or warfarin. Baseline characteristics were balanced using inverse probability of treatment weighting with stabilised weights (s-IPTW). Bleeding risk and stroke/SE risk were expressed as HRs with 95% CIs. Two sensitivity analyses were conducted.

**Results:**

A total of 73 989 patients were eligible for analysis. Notably, 52.8%–81.9% of patients received reduced doses of NOACs. After applying s-IPTW, patient characteristics were well balanced across warfarin/NOAC cohorts. The mean within-cohort age, CHADS_2_ score and CHA_2_DS_2_-VASc score were 76 years, 2.2–2.3 and 3.8, respectively. In all age categories, the majority of the HRs for major bleeding, any bleeding and stroke/SE were equal to or below 1 for all NOACs versus warfarin. Apixaban was the only NOAC associated with a significantly lower risk of any bleeding. There was a trend towards increased risk reduction with NOACs versus warfarin in patients with body weight ≥60 kg. In patients with renal disease, the HRs for apixaban versus warfarin were below 1 for major bleeding, any bleeding and stroke/SE, with statistical significance observed for the risk reduction in stroke/SE versus warfarin. In the sensitivity analysis, there were no large differences in HRs between the two observational periods.

**Conclusions:**

In patients with NVAF primarily treated with reduced-dose NOACs, the risks of stroke/SE and major bleeding were significantly lower with NOACs versus warfarin.

**Key questions**

**What is already known about this subject?**

- For the prevention of stroke and systemic embolism (SE) in patients with non-valvular atrial fibrillation (NVAF), clinical guidelines recommend treatment with non-vitamin K oral anticoagulants (NOACs) rather than warfarin. However, the effectiveness and safety of NOACs in Japanese clinical practice remain to be fully elucidated, particularly in patients with high-risk profiles compared with those enrolled in clinical trials.

**What does this study add?**

- This study found that the majority of patients with NVAF treated in Japanese clinical practice received reduced doses of NOACs—a treatment pattern likely underpinned by bleeding-related concerns. Despite the dose reduction, the risks of stroke/SE, major bleeding and major intracranial haemorrhage were significantly lower for NOACs versus warfarin in Japanese patients with NVAF.

**How might this impact on clinical practice?**

- These findings provide important real-world evidence describing treatment patterns and clinical outcomes for elderly patients with NVAF treated in Japanese clinical practice. They indicate that NOAC treatment was associated with clinical benefits versus warfarin, even in a population primarily treated with reduced doses.

## Introduction

Atrial fibrillation (AF) is the most common arrhythmia and is observed in <1% of the total population in Japan.^1^ The prevalence of AF increases with age, rising to approximately 14% in patients aged >80 years.^1 2^ AF is a well-established risk factor for stroke, systemic embolism (SE) and death.^3 4^ Recent guidelines recommend treatment with non-vitamin K oral anticoagulants (NOACs) (ie, apixaban, dabigatran, edoxaban and rivaroxaban) for eligible oral anticoagulant (OAC)-naïve patients with non-valvular atrial fibrillation (NVAF).^2 5^ Multiple randomised controlled trials (RCTs) have supported the benefits of NOACs versus warfarin in patients with NVAF,^6–9^ with a meta-analysis confirming that NOACs significantly lower the risk of stroke/SE with a risk of major bleeding similar to that associated with warfarin.^10^

While RCTs are the gold standard for demonstrating the effectiveness of interventions, they are not fully representative of an unselected real-world population, thereby limiting the relevance of their findings to clinical practice. Consequently, a number of observational, real-world evidence studies have emerged to provide supportive evidence of the safety and/or effectiveness of NOACs in clinical practice.^11–18^ However, there remain several unmet knowledge gaps in the literature regarding the clinical outcomes of NOAC treatment in patients with NVAF, particularly in patient subgroups at high risk of adverse outcomes.^19 20^

All four NOACs (apixaban, dabigatran, edoxaban and rivaroxaban) have been approved in Japan for the prevention of stroke and SE in patients with NVAF.^21^ Importantly, dosing of NOACs in Japan differs slightly from that in other countries given the higher bleeding complication rates reported in East Asian patients; for example, the approved dose of rivaroxaban is 10/15 mg daily in Japan.^21^ Given the unique setting surrounding the use of NOACs, and considering they are often initiated at reduced doses, the impact of NOACs on safety (ie, the risk of bleeding) and effectiveness (ie, the risk of stroke or SE) outcomes in Japanese patients with NVAF requires further elucidation.

## Methods

### Study design

This was a non-interventional, retrospective, observational study conducted from March 2011 (ie, when the first NOAC, dabigatran, was approved in Japan) to July 2018 to evaluate the safety and effectiveness of apixaban, dabigatran, edoxaban and rivaroxaban, each separately, versus warfarin in Japanese patients with NVAF. Written consent from study participants was not necessary in a retrospective study using an existing structured database according to the Japanese Ethical Guidelines. All data were anonymised, and any information that could be used to identify individuals or hospitals was removed.

We used deidentified health claims data from 372 acute care hospitals across Japan available from the Medical Data Vision Co Ltd (MDV; Tokyo, Japan) database.^22^ In brief, the MDV database comprises administrative data for approximately 24 million individuals in the inpatient and outpatient settings.^22^ Each patient is assigned a specific ID to which all inpatient and outpatient data are linked. The distribution of demographic characteristics, including age and sex, of patients registered in the MDV database is very similar to the national population statistics in Japan. For each prescription recorded in the MDV database, the diagnosis is listed according to 10th Revision of the International Classification of Diseases (ICD-10) codes or local disease codes.

Patients registered in the MDV database between 1 March 2011 and 31 July 2018 were selected based on the following inclusion criteria: diagnosis of AF at any time during the preindex period and first prescription of any OAC (apixaban, dabigatran, edoxaban, rivaroxaban or warfarin) after a diagnosis of AF; age 18 years or older on the index date (defined as the date of the first prescription of any OAC); and no OAC prescription during the year preceding the index date (baseline period). The first OAC prescription recorded in the database was used to identify the patient’s index date, treatment cohort and OAC dose. Patients with a diagnosis of valvular AF, postoperative AF, AF associated with mechanical valve malfunction, AF associated with mechanical complication of heart valve prosthesis or rheumatic AF during the baseline period were excluded. Additionally, patients with a diagnosis of hyperthyroidism or thyrotoxicosis, those who underwent procedures involving prosthetic heart valves performed during the baseline period and those with haemodialysis or pregnancy during the baseline period were also excluded.

Patients were followed from the index date until any of the following events, whichever occurred first: discontinuation of the index OAC, defined as a continuous gap of 45 days or more between the expected refill date and the actual refill date; switch to another OAC—if the index OAC was discontinued and another OAC was started within 45 days of the prescription refill date of the index OAC; lack of further records in the database—if no further relevant records were added (eg, no further refills or visits), the last date of the patient’s record in the database was used; occurrence of stroke, SE or haemorrhagic adverse events; or an elapse of 2 years from the index date.

### Endpoints

Individual NOACs and warfarin were compared with respect to the incidence of stroke/SE and bleeding in cohorts after inverse probability of treatment weighting with stabilised weights (s-IPTW) was applied. The safety endpoints were major bleeding and any bleeding, defined as bleeding requiring hospitalisation (major bleeding) and any bleeding event recorded after the index date regardless of severity or need for hospitalisation (any bleeding). Bleeding sites were not considered in the primary analysis. The effectiveness primary endpoint was a composite of stroke and SE requiring hospitalisation. Stroke was defined as ischaemic or haemorrhagic stroke. Haemorrhagic stroke was included both as a safety endpoint and as an effectiveness endpoint. For the secondary analyses, major gastrointestinal (GI) bleeding, any GI bleeding, major intracranial haemorrhage (ICH) and any ICH were the safety-related secondary endpoints. Ischaemic stroke, haemorrhagic stroke and SE were the effectiveness-related secondary endpoints. The primary safety and effectiveness endpoints were also assessed in the following prespecified subgroups: age (≥75 years/<75 years or ≥80 years/<80 years), body weight (≥60 kg/<60 kg), renal disease (yes/no), concomitant use of antiplatelet drugs (yes/no) and NOAC dose (standard/reduced). Similar to the primary analyses, s-IPTW was applied to balance patient characteristics among these subgroups. The ICD-10 codes and disease codes used in the study are listed in online supplementary tables 1 and 2.

10.1136/openhrt-2019-001232.supp1 Supplementary data

### Statistics

All analyses were conducted with SAS V.9.4. A propensity score was calculated based on multinomial logistic regression in order to account for confounding effects and to ensure that patient characteristics were balanced between the NOAC and warfarin cohorts. An IPTW method using the calculated propensity score was applied, and to avoid sample size inflation and ensure appropriate estimation of variances, s-IPTW was used.^23 24^ Weight truncation was not conducted. The following clinical and demographic characteristics, collected during the baseline period or at the index date, were included as covariates to calculate the propensity score: sex and age, comorbidities (ie, heart failure, coronary heart disease, peripheral vascular disorder, myocardial infarction, stroke, transient ischaemic attack, SE, renal dysfunction, hepatic dysfunction, bleeding history, hypertension and diabetes mellitus), concomitant medications (ie, antiplatelet drugs, nonsteroidal anti-inflammatory drugs, gastric secretion inhibitors, statins, heparins and antihypertensive drugs) and presence of cardioversion and ablation procedures. CHADS_2_ and CHA_2_DS_2_-VASc scores were calculated using these clinical and demographic characteristics.^25 26^ The calculated s-IPTW was simultaneously applied to the five crude OAC cohorts to obtain four paired NOAC/warfarin cohorts, wherein demographic and clinical characteristics of each OAC cohort were balanced. The covariate balance between the NOAC/warfarin cohorts after s-IPTW was assessed with respect to standardised differences using a threshold of 0.1; previous studies have suggested that a standardised difference of >0.1 may indicate the presence of a meaningful imbalance of covariates between paired treatments.^27–29^ The 2-year cumulative incidence rates of major bleeding, any bleeding and stroke/SE in the cohorts after s-IPTW were plotted with Kaplan-Meier curves. HRs with 95% CIs were calculated using a Cox proportional hazards regression model that incorporated only the index OACs as independent variables.

### Sensitivity analyses

Two sensitivity analyses were conducted. First, a sensitivity analysis was performed by restricting the follow-up period to 1 year, and differences in the results versus the 2-year follow-up period were compared. Second, a conventional 1:1 propensity score matching method was used to assess the robustness of the method used for addressing the covariate imbalance between cohorts. As in the main analysis, a threshold of 0.1 was used for confirming covariate balance between the two groups, and HRs with 95% CIs were calculated using a Cox proportional hazards model.

## Results

### Baseline characteristics in the crude cohorts before s-IPTW

**Figure 1:**
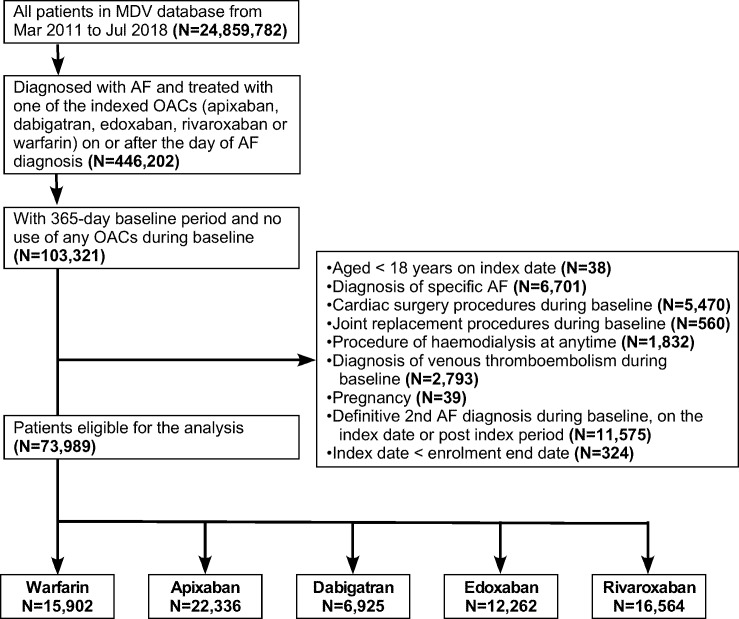
Flow chart of patient allocation to each OAC cohort. AF, atrial fibrillation; MDV, Medical Data Vision Co Ltd; OAC, oral anticoagulant.

Overall, 73 989 patients were eligible for the analysis after applying the selection criteria (figure 1). Patients were divided into five cohorts: 15 902 patients initiated warfarin; 22 336 patients initiated apixaban 2.5 mg or 5 mg twice daily; 6925 patients initiated dabigatran 110 mg or 150 mg twice daily; 12 262 patients initiated edoxaban 30 mg or 60 mg once daily; and 16 564 patients initiated rivaroxaban 10 mg or 15 mg once daily (figure 1). Baseline characteristics in the crude cohorts before s-IPTW are reported in online supplementary table S3. The mean (SD) duration of treatment ranged from 265 (263.8) to 868 (725) days. Apixaban was the most frequently prescribed NOAC, and 47.2%–76.2% of patients were initiated on reduced doses of NOACs. The warfarin cohort contained the oldest patients, with the highest mean CHADS_2_ and CHA_2_DS_2_-VASc scores and the most comorbidities, and patients in the apixaban cohort tended to be older with higher mean risk scores (online supplementary table S3).

### Incidence of bleeding and stroke/SE in the crude cohorts before s-IPTW

Between-cohort differences in event rates (per 100 person-years) reflected differences in baseline patient risk characteristics (online supplementary table S3). The event rates of major bleeding, any bleeding and stroke/SE were highest in the warfarin cohort; the event rates of major bleeding and any bleeding were lowest in the dabigatran cohort, and the event rate of stroke/SE was lowest in the rivaroxaban cohort (online supplementary table S4).

### Patient characteristics in the cohorts after s-IPTW

The standardised differences between the s-IPTW-balanced cohorts in patient characteristics used for calculating the propensity score were <0.1, suggesting that patient characteristics were well balanced between the cohorts (table 1). For patients treated with dabigatran, the age, CHADS_2_ and CHA_2_DS_2_-VASc scores and proportion of patients with comorbidities were slightly higher than those in the crude cohort. The proportion of patients treated with reduced doses of NOACs remained high (52.8%–81.9%; table 1).

**Table 1:** Demographics and clinical characteristics of patients with 365-day baseline after s-IPTW

Variables	Warfarin n=19 059	Apixaban 5/2.5 mg twice daily n=22 752		Dabigatran 150/110 mg twice daily n=8003		Edoxaban 60/30 mg once daily n=12 592		Rivaroxaban 15/10 mg once daily n=17 481	
	N (%)	N (%)	Std. diff.*	N (%)	Std. diff.*	N (%)	Std. diff.*	N (%)	Std. diff.*
Sex category (male)†	11 656 (61.2)	13 912 (61.2)	−0.0002	4963 (62.0)	0.0176	7691 (61.1)	−0.0018	10 672 (61.1)	−0.0022
Age, years									
Mean±SD†	76.1±11.9	76.1±10.8	−0.0016	75.6±10.3	−0.0464	76.2±10.8	0.0048	76.2±10.6	0.0074
<65	2542 (13.3)	2829 (12.4)	−0.0270	954 (11.9)	−0.0428	1586 (12.6)	−0.0222	2119 (12.1)	−0.0365
65–74	4707 (24.7)	5984 (26.3)	0.0368	2330 (29.1)	0.0996	3249 (25.8)	0.0254	4679 (26.8)	0.0473
≥75	11 809 (62.0)	13 939 (61.3)	−0.0144	4719 (59.0)	−0.0612	7757 (61.6)	−0.0074	10 683 (61.1)	−0.0175
BMI, kg/m^2^									
<18	1170 (6.1)	1048 (4.6)	−0.0679	286 (3.6)	−0.1193	614 (4.9)	−0.0553	878 (5.0)	−0.0486
18 to <22	3925 (20.6)	3555 (15.6)	−0.1293	1123 (14.0)	−0.1741	1858 (14.8)	−0.1536	2587 (14.8)	−0.1524
22 to <25	3541 (18.6)	3123 (13.7)	−0.1322	1185 (14.8)	−0.1012	1572 (12.5)	−0.1690	2356 (13.5)	−0.1394
≥25	3193 (16.8)	2671 (11.7)	−0.1438	1024 (12.8)	−0.1117	1320 (10.5)	−0.1837	2181 (12.5)	−0.1213
Missing	7230 (37.9)	12 354 (54.3)	0.3328	4384 (54.8)	0.3428	7229 (57.4)	0.3975	9479 (54.2)	0.3313
Dose group									
Reduced	–	12 539 (55.1)	NA	6557 (81.9)	NA	9376 (74.5)	N/A	9221 (52.8)	N/A
Standard	–	10 213 (44.9)	NA	1445 (18.1)	NA	3216 (25.5)	N/A	8260 (47.3)	N/A
CHADS_2_									
Mean±SD	2.3±1.6	2.3±1.5	−0.0216	2.2±1.6	−0.0466	2.3±1.6	−0.0176	2.3±1.5	−0.0211
0	2003 (10.5)	2914 (12.8)	0.0715	1007 (12.6)	0.0650	1648 (13.1)	0.0800	2107 (12.1)	0.0487
1	4517 (23.7)	5246 (23.1)	−0.0152	1861 (23.3)	−0.0105	2889 (22.9)	−0.0180	4089 (23.4)	−0.0073
2	4511 (23.7)	4863 (21.4)	−0.0550	1858 (23.2)	−0.0106	2669 (21.2)	−0.0594	3881 (22.2)	−0.0349
≥3	8028 (42.1)	9730 (42.8)	0.0130	3276 (40.9)	−0.0240	5387 (42.8)	0.0133	7404 (42.4)	0.0047
CHA_2_DS_2_-VASc									
Mean±SD	3.8±2.1	3.8±1.9	−0.0146	3.8±2.0	−0.0346	3.8±2.0	−0.0124	3.8±1.9	−0.0127
0	574 (3.0)	783 (3.4)	0.0241	236 (3.0)	−0.0037	455 (3.6)	0.0335	520 (3.0)	−0.0023
1	1408 (7.4)	1732 (7.6)	0.0085	656 (8.2)	0.0302	1017 (8.1)	0.0259	1328 (7.6)	0.0079
2	2783 (14.6)	3578 (15.7)	0.0312	1239 (15.5)	0.0246	1965 (15.6)	0.0280	2732 (15.6)	0.0286
≥3	14 293 (75.0)	16 660 (73.2)	−0.0404	5871 (73.4)	−0.0372	9155 (72.7)	−0.0522	12 901 (73.8)	−0.0273
PT-INR‡ (mean±SD)	1.60±0.74	–	–	–	–	–	–	–	–
Heart failure†	7156 (37.5)	8442 (37.1)	−0.0091	2944 (36.8)	−0.0158	4679 (37.2)	−0.0080	6480 (37.1)	−0.0098
Coronary heart disease†	4873 (25.6)	5808 (25.5)	−0.0009	2041 (25.5)	−0.0015	3198 (25.4)	−0.0040	4407 (25.2)	−0.0082
Peripheral arterial disorder†	1447 (7.6)	1710 (7.5)	−0.0028	606 (7.6)	−0.0007	940 (7.5)	−0.0047	1312 (7.5)	−0.0033
Myocardial infarction†	570 (3.0)	676 (3.0)	−0.0013	238 (3.0)	−0.0013	370 (2.9)	−0.0033	516 (3.0)	−0.0024
Hyperthyroidism or thyrotoxicosis	434 (2.3)	477 (2.1)	−0.0121	172 (2.1)	−0.0089	264 (2.1)	−0.0120	362 (2.1)	−0.0139
Stroke, TIA or SE†	4086 (21.4)	4756 (20.9)	−0.0131	1624 (20.3)	−0.0280	2641 (21.0)	−0.0113	3696 (21.2)	−0.0071
Renal dysfunction†	1326 (7.0)	1554 (6.8)	−0.0051	560 (7.0)	0.0014	864 (6.9)	−0.0039	1224 (7.0)	0.0017
Liver dysfunction†	2454 (12.9)	2857 (12.6)	−0.0096	1034 (12.9)	0.0013	1583 (12.6)	−0.0092	2184 (12.5)	−0.0116
Bleeding diagnosis†	2322 (12.2)	2754 (12.1)	−0.0025	1002 (12.5)	0.0101	1530 (12.2)	−0.0011	2136 (12.2)	0.0010
Hypertension†	10 650 (55.9)	12 527 (55.1)	−0.0165	4377 (54.7)	−0.0238	6945 (55.2)	−0.0147	9602 (54.9)	−0.0191
Diabetes mellitus†	5791 (30.4)	6833 (30.0)	−0.0077	2414 (30.2)	−0.0049	3778 (30.0)	−0.0085	5236 (30.0)	−0.0095
Cancer	4201 (22.0)	5252 (23.1)	0.0249	1872 (23.4)	0.0323	2969 (23.6)	0.0367	3925 (22.5)	0.0099
Treated with antiplatelet drugs†	4459 (23.4)	5181 (22.8)	−0.0149	1781 (22.3)	−0.0272	2870 (22.8)	−0.0145	3960 (22.7)	−0.0177
Treated with NSAIDs†	5947 (31.2)	6993 (30.7)	−0.0102	2474 (30.9)	−0.0062	3894 (30.9)	−0.0061	5401 (30.9)	−0.0067
Treated with gastric secretion inhibitor†	7736 (40.6)	9113 (40.1)	−0.0110	3180 (39.7)	−0.0175	5045 (40.1)	−0.0108	7022 (40.2)	−0.0086
Treated with statin-based drug†	2677 (14.0)	3200 (14.1)	0.0006	1084 (13.5)	−0.0146	1765 (14.0)	−0.0008	2410 (13.8)	−0.0074
Treated with antiarrhythmics	8481 (44.5)	9968 (43.8)	−0.0138	3512 (43.9)	−0.0125	5529 (43.9)	−0.0120	7677 (43.9)	−0.0117
Treated with beta-blockers	3972 (20.8)	4651 (20.4)	−0.0099	1613 (20.2)	−0.0169	2569 (20.4)	−0.0110	3526 (20.2)	−0.0167
Treated with heparin†	3905 (20.5)	4552 (20.0)	−0.0121	1625 (20.3)	−0.0048	2520 (20.0)	−0.0119	3555 (20.3)	−0.0039
Cardioversion†	142 (0.7)	162 (0.7)	−0.0038	67 (0.8)	0.0101	91 (0.7)	−0.0030	122 (0.7)	−0.0052
Therapy days (mean±SD)	451.5±632.9	395.9±412.1	−0.1041	823.4±765.1	0.5296	263.0±266.7	−0.3882	415.4±471.2	−0.0647

*Calculated when compared with the warfarin cohort.

†Variables included in the calculation of propensity score.

‡The Japanese treatment guidelines recommend target INR ranges of 2.0–3.0 for patients aged less than 70 years and 1.6–2.6 for patients aged 70 years or older.

BMI, body mass index; NSAID, non-steroidal anti-inflammatory drug; PT-INR, prothrombin time–international normalised ratio; SE, systemic embolism; s-IPTW, inverse probability of treatment weighting with stabilised weights; TIA, transient ischaemic attack.

### Bleeding and stroke/SE risk in the cohorts after s-IPTW

**Figure 2:**
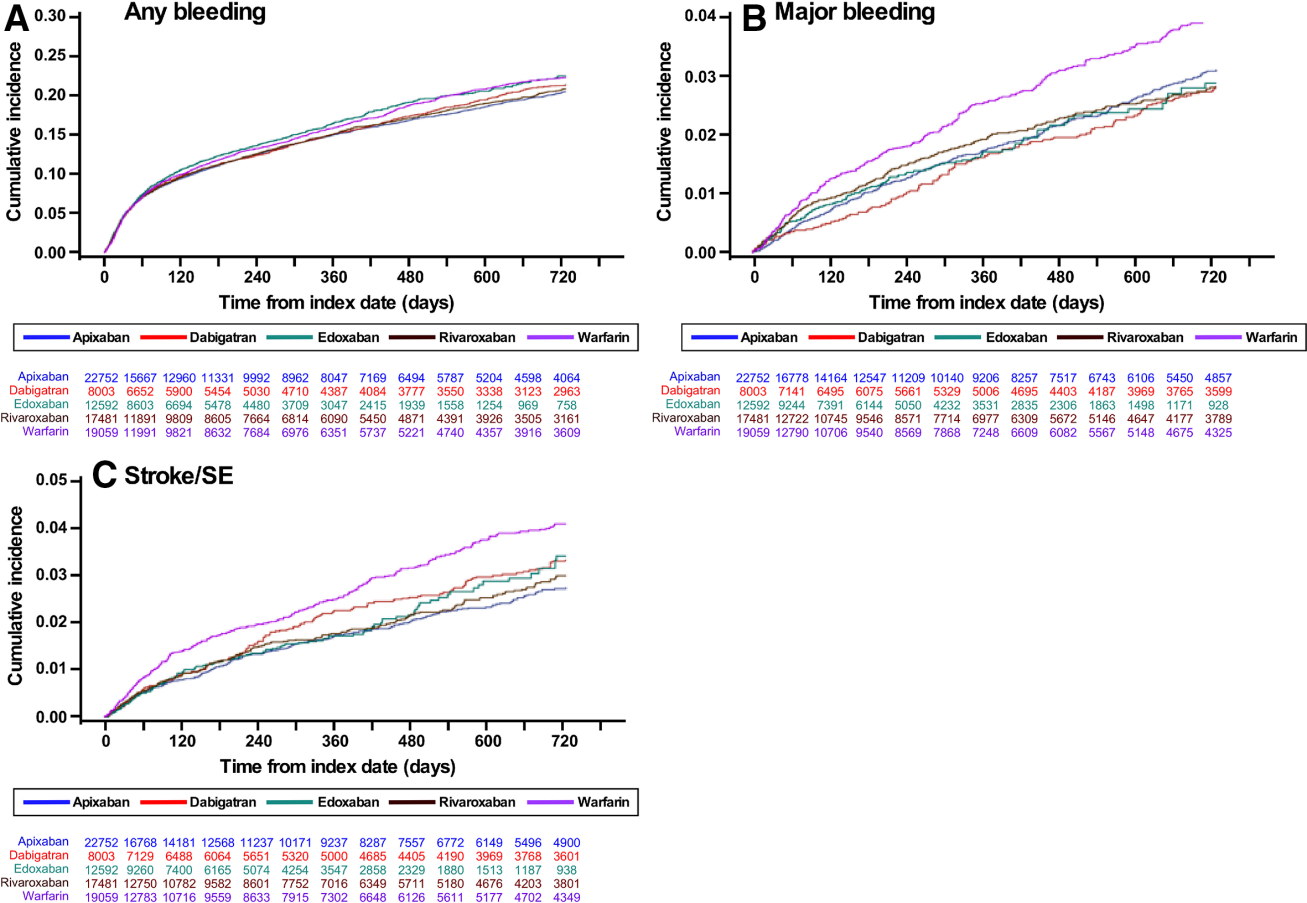
Kaplan-Meier curves for incidence of (A) any bleeding, (B) major bleeding and (C) stroke/SE. SE, systemic embolism.

**Figure 3:**
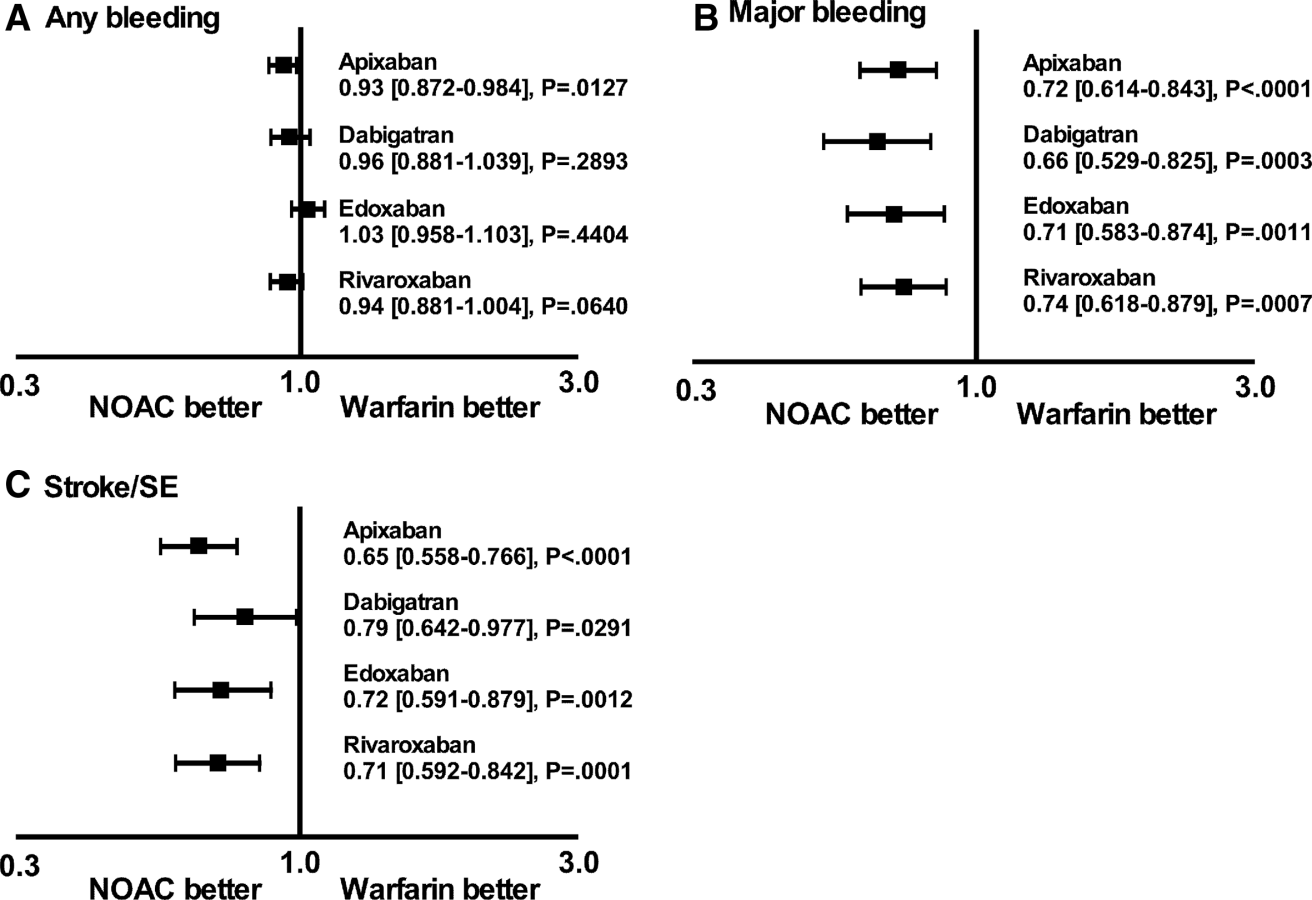
Forest plot depicting the risk of events for NOACs versus warfarin. HRs and 95% CIs are given for each NOAC. NOAC, non-vitamin K oral anticoagulant; SE, systemic embolism.

Unweighted Kaplan-Meier cumulative incidence plots of any bleeding, major bleeding and stroke/SE events are presented in figure 2. Compared with warfarin, all NOACs were associated with a significantly lower risk of stroke/SE and major bleeding (figure 3). Apixaban was associated with a significantly lower risk of any bleeding, and dabigatran and rivaroxaban had HRs below 1; however, statistical significance was not achieved (figure 3).

### Secondary safety and effectiveness endpoints

A significantly lower risk of major ICH was observed for all NOACs versus warfarin, and dabigatran and rivaroxaban were associated with a significantly lower risk of any ICH (table 2). Apixaban was associated with a significantly lower risk of major GI bleeding, and apixaban and rivaroxaban were associated with a significantly lower risk of any GI bleeding (table 2). Compared with warfarin, all of apixaban, edoxaban and rivaroxaban were associated with a significantly lower risk of ischaemic stroke, while dabigatran and rivaroxaban were associated with a significantly lower risk of haemorrhagic stroke. All NOACs had HRs below 1 for SE; however, statistical significance was not achieved (table 2).

**Table 2:** HRs with 95% CIs for secondary endpoints

	N (NOAC/warfarin)	Apixaban 5/2.5 mg twice daily over warfarin	Dabigatran 150/110 mg twice daily over warfarin	Edoxaban 60/30 mg once daily over warfarin	Rivaroxaban 15/10 mg once daily over warfarin
		22 752/19 059	8 003/19 059	12 592/19 059	17 481/19 059
Ischaemic stroke	HR	0.63	0.90	0.74	0.74
	95% CI	(0.524 to 0.759)	(0.716 to 1.140)	(0.586 to 0.925)	(0.607 to 0.909)
	P value	<0.0001	0.3906	0.0087	0.0039
Haemorrhagic stroke	HR	0.75	0.41	0.73	0.63
	95% CI	(0.545 to 1.029)	(0.244 to 0.703)	(0.479 to 1.102)	(0.432 to 0.922)
	P value	0.0743	0.0011	0.1332	0.0175
Systemic embolism	HR	0.48	0.97	0.46	0.50
	95% CI	(0.198 to 1.165)	(0.316 to 2.959)	(0.145 to 1.487)	(0.183 to 1.349)
	P value	0.1050	0.9519	0.1966	0.1701
Major GI bleeding	HR	0.76	0.92	0.83	0.92
	95% CI	(0.579 to 0.987)	(0.655 to 1.286)	(0.602 to 1.158)	(0.693 to 1.213)
	P value	0.0394	0.6175	0.2798	0.5425
Any GI bleeding	HR	0.87	1.04	0.99	0.87
	95% CI	(0.779 to 0.970)	(0.901 to 1.203)	(0.871 to 1.125)	(0.768 to 0.976)
	P value	0.0121	0.5870	0.8812	0.0186
Major intracranial haemorrhage	HR	0.58	0.42	0.60	0.52
	95% CI	(0.452 to 0.757)	(0.283 to 0.637)	(0.427 to 0.836)	(0.379 to 0.702)
	P value	<0.0001	<0.0001	0.0026	<0.0001
Any intracranial haemorrhage	HR	0.89	0.79	0.92	0.81
	95% CI	(0.781 to 1.010)	(0.658 to 0.946)	(0.789 to 1.076)	(0.701 to 0.936)
	P value	0.0715	0.0104	0.3014	0.0044

GI, gastrointestinal; NOAC, non-vitamin K oral anticoagulant.

### Subgroup analyses in patients with high-risk profiles

Online supplementary table S5 reports the results of the subgroup analysis in high-risk patients. Across age categories, the majority of the HRs for major bleeding, any bleeding and stroke/SE were equal to or below 1 for all NOACs versus warfarin, although not all were statistically significant. In the very elderly age group (≥80 years), apixaban, edoxaban and rivaroxaban were associated with a significantly lower risk of major bleeding and stroke/SE (online supplementary table S5).

In patients with body weight <60 kg, apixaban was associated with a significantly lower risk of stroke/SE, and there was a trend towards risk reduction for major bleeding and any bleeding with NOACs versus warfarin in patients with body weight ≥60 kg. In patients with renal disease, the HRs for apixaban alone (vs warfarin) were below 1 for major bleeding, any bleeding and stroke/SE, with statistical significance observed for the risk reduction in stroke/SE versus warfarin (online supplementary table S5).

When stratified by initial dose (ie, standard vs reduced), the risk of any bleeding was significantly higher with the standard dose of edoxaban, and the risk of stroke/SE was significantly lower with a reduced dose of apixaban versus warfarin (online supplementary table S5).

### Sensitivity analysis

There were no large differences in HRs between the two different observational periods (1 year and 2 years), although statistical significance was not always obtained for the HRs in the 1-year observation period owing to the small number of events (online supplementary table S6). Results of the second sensitivity analysis were also largely consistent with the main results (online supplementary table S7).

## Discussion

In this large, real-world, observational study, we evaluated the effectiveness and safety of four NOACs currently approved for stroke/SE prevention versus warfarin in Japanese patients with NVAF. The primary results indicated that all NOACs were associated with a significantly lower risk of major bleeding and stroke/SE compared with warfarin. Notable results from the secondary analyses were a significantly lower risk of major ICH for all NOACs, and reductions in the risk of any and major GI bleeding with apixaban, versus warfarin. Broadly, these real-world results provide supportive evidence for the existing RCTs that have demonstrated the clinical benefits of NOACs versus warfarin in patients with NVAF.^6–10^ Moreover, the current study builds on the emerging, real-world evidence base for the effectiveness and safety of NOACs in Japanese clinical practice.^15–18 30^

Reduced dosing of NOACs is a pertinent clinical concern as it may impact the safety and/or effectiveness of treatment.^18 31 32^ In the current study, risks for bleeding and stroke/SE were generally consistent between the standard-dose and reduced-dose NOAC subgroups, and we observed a significantly lower risk of stroke/SE with reduced-dose apixaban versus warfarin. Thus, the current results differ from recent real-world study results, in which reduced-dose NOAC treatment was associated with increased rates of thromboembolic and major haemorrhagic events, along with stroke/SE and myocardial infarction, in Japanese patients with NVAF.^18 32^ Of note, the proportions of patients initiated on reduced doses of NOACs were higher than those reported in studies conducted in the USA,^33–35^ Korea and Taiwan^36–39^ and in real-world studies in Japan.^17 18 40^ It is likely that the high rates of dose reduction observed in the current study were primarily attributable to the risk characteristics of the patient sample. For instance, in a recent cross-sectional analysis of a multicentre outpatient registry in Japan, the independent predictors of NOAC underdosing in newly diagnosed patients with NVAF were older age, concomitant antiplatelet therapy, impaired renal function and prior heart failure or left ventricular dysfunction.^40^

Appropriate use of NOACs in patients with NVAF and comorbid renal disease remains the subject of ongoing investigation,^41–45^ and worsening of renal function in patients with AF is independently associated with ischaemic stroke and haemorrhage.^43^ NOAC-specific differences in renal excretion rates have been observed,^44 45^ with dabigatran and edoxaban having the greatest dependence on renal elimination compared with apixaban and rivaroxaban.^45^ A recent meta-analysis of RCTs and observational studies in patients with renal disease reported that NOACs significantly lowered the risk of ICH, stroke/SE and major bleeding versus warfarin.^42^ In the current study, apixaban was the only NOAC with a significantly lower risk of stroke/SE, whereas the risk of stroke/SE was significantly higher for rivaroxaban versus warfarin in patients with NVAF and comorbid renal disease. However, owing to the relatively small number of patients with renal disease, along with the observational design of the study, firm conclusions regarding the safety and effectiveness of NOACs in Japanese patients with NVAF and comorbid renal disease should not be made on the basis of these results.

Strengths of the current study’s design and results include the MDV database being representative of the Japanese population, the high mean age of the patients (ie, most were very elderly), the large sample size and the inclusion of all four approved NOACs in the analyses. Additionally, a majority of the patients were treated with reduced doses of NOACs, which allowed for the evaluation of effectiveness and safety in patients on reduced doses; however, this also places limitations on the generalisability of the results to patients with NVAF primarily treated with standard doses of NOACs. Furthermore, the study provides much-needed data on the effectiveness and safety of NOACs in Japanese patients with NVAF, as many studies have been conducted in Western populations. However, the study has several limitations. First, data were obtained from a claims database containing information provided by hospitals applying the flat-fee payment system, which are mostly large hospitals responsible for acute care. Therefore, a significant proportion of the patients included were likely in poorer health than the average population requiring hospitalisation, possibly having more comorbidities and a higher risk of stroke/SE and bleeding. Second, the claims data did not include vital signs or laboratory measurements (eg, blood pressure, international normalised ratio values, renal function parameters), which precluded calculation of a HAS-BLED score.^46^ Therefore, we were unable to consider these variables in the calculation of the propensity score, and consequently, there is no guarantee that these characteristics were fully balanced after s-IPTW. Thus, the influence of unexamined confounding factors cannot be fully excluded. Third, we could not provide an estimate of follow-up loss as we had no subsequent data on patients who had visited a different hospital or clinic after being registered with one of the hospitals contributing to the MDV database. This could have led to an underestimation of the incidence of stroke/SE or major bleeding events. Fourth, results of some subgroup analyses are not conclusive owing to the smaller number of patients and lower statistical power. Fifth, primary endpoints were defined as resulting in hospitalisation, which differs from the adjudicated endpoints typically used in RCTs, and we did not include a mortality endpoint. Finally, while the majority of patients received reduced doses of NOACs, we were unable to determine whether this level of dosing was appropriate or if off-label underdosing of NOACs had any impact on the clinical outcomes owing to the unavailability of clinical information in the MDV database.

In conclusion, a large proportion of patients with NVAF initiated treatment with reduced-dose NOACs in contemporary Japanese practice. Despite this, the risks of stroke/SE, along with major bleeding, were significantly lower for NOACs versus warfarin. The results were largely consistent across the patient subgroups with higher risk profiles, such as those with older age, lower body weight and renal disease.
