# Supplementary material for: Safety and effectiveness of non-vitamin K oral anticoagulants versus warfarin in real-world patients with non-valvular atrial fibrillation: a retrospective analysis of contemporary Japanese administrative claims data
Source: Open Heart. 2020 Apr 1;7(1):e001232. doi: 10.1136/openhrt-2019-001232 (PMC7174060; doi:10.1136/openhrt-2019-001232)
Supplement: Supplementary data [file openhrt-2019-001232supp001.pdf]

Supplemental Materials

Table S1. Definitions of bleeding, stroke, and systemic embolism by ICD-10 codes and/or standard disease codes

**Diseases or comorbid conditions**	**ICD-10 code and/or standard disease code**
Bleeding	A162* (*8833037*[†]), A165 (*8833043*), B303 (*8832369*), D500 (*2809005*), D62 (*8832363, 8835357*), D66 (*8833200*), D683 (*2865003*), D698 (*2878003, 4489007, 8840572*), D699 (*2869010, 2872016*), E078 (*8833510*), E274 (*8839683*), G361 (*8830185, 8832371*), G951 (*3361005, 3361013, 3361019, 4320004, 8847133*), G968 (*8835981*), H052 (*3763004*), H113 (*3727006, 8831968*), H168 (*8834634*), H208 (*8834639*), H210 (*3644003, 8836580*), H313 (*3636002, 3636003, 8840453*), H350 (*3621012, 8834644*), H356 (*3628002, 3628013, 3628018, 3628019, 3628022, 3628025, 8830975, 8834734, 8840624, 8840631, 8840635*), H357 (*8847483*), H405 (*3659001*), H431 (*3792006, 8842347*), H448 (*3604008*), H470 (*8834333, 8834344*), H603 (*8834635*), H669 (*8834642*), H738 (*3848003*), H922 (*3809001, 3888004*), I213 (*8847041*), I230 (*8832383*), I312 (*8835136, 8835137*), I600 (*8838299, 8846321, 8847449, 8847541*), I601 (*8837619, 8847527*), I602 (*8836504, 8847505*), I603 (*8833433, 8847468*), I604 (*8838740, 8847545*), I605 (*8837825, 8847531*), I606 (*8846593, 8846750, 8847469, 8847506*), I607 (*8847536*), I608 (*8835797, 8842228, 8842229, 8847895, 8847896*), I609 (*4309001, 4309005, 8836339, 8838751*), I610 (*4310038, 4319027, 4319030, 8847680*), I611 (*8839257*), I613 (*4319013, 8841358, 8845147*), I614 (*4319006*), I615 (*4319018, 4319032*), I616 (*836998*), I618 (*4320007*), I619 (*4319003, 4319009, 4319020, 8847897*), I620 (*4321006, 4321008, 8833690, 8843499*), I621 (*8843500*), I629 (*8839202*), I638 (*4341044*), I690 (*4389001*), I691 (*4389017*), I780 (*4480004, 8848472*), I788 (*4489012*), I841 (*8834643*), I844 (*8834636*), I848 (*8834640*), I850 (*4560002, 4560003*), I864 (*8845850*), J041 (*8834638*), J339 (*4709003*), J90 (*8833110*), J942 (*5118002, 5118003*), J950 (*8832181*), K049 (*5220084*), K068 (*8834466*), K092 (*8834637*), K121 (*5280051*), K137 (*5289010*), K148 (*8836434*), K226 (*8840294*), K228 (*5308005*), K250 (*5319011, 5350009, 8847768, 8847779, 8847799*), K252 (*8845122*), K254 (*8834632, 8847788, 9620001*), K256 (*8845130*), K260 (*8845123, 8847773, 8847780*), K262 (*8845124*), K264 (*8834641*), K266 (*8845131*), K270 (*8847744, 8847769, 8847778*), K284 (*8847762*), K290 (*8834631*), K571 (*8845800*), K573 (*8845742, 8845749, 8845763, 8845806, 8845814*), K625 (*5693001, 5789011, 8833703*), K661 (*8839651, 8839763, 8841643*), K762 (*8837584*), K768 (*8831551*), K85 (*8832368, 8832370*), K920 (*5780005*), K921 (*5781001, 5781002, 5789005, 7921002*), K922 (*5780004, 5789001, 5789007, 5789008, 837732, 8848141*), L508 (*7573086*), M2506 (*9241024*), M2509 (*8831595*), N029 (*5997004, 5997005, 5997012, 5997016, 8833171, 8834239, 8841019, 8841023*), N288 (*5938021, 5938028, 8835604*), N300 (*5950002*), N304 (*8847564*), N309 (*8842024*), N328 (*5967004*), N368 (*5997011*), N421 (*6021002*), N488 (*6078036*), N501 (*8830697, 8835847*), N645 (*6117024, 6117032*), N830 (*6201005, 8834645*), N831 (*6201001, 8834633*), N836 (*8840898*), N837 (*8834223*), N838 (*6201004*), N898

Diseases or comorbid conditions	ICD-10 code and/or standard disease code
	(*8837499*), N908 (*6245002*), N921 (*6268001, 8834262*), N922 (*6263003, 6263005*), N923 (*6265001, 6265002*), N924 (*6271002*), N930 (*8835843*), N938 (*6263004, 8832247*), N939 (*6268005, 6269007*), N950 (*8839932*), O717 (*6245003, 6645001, 6659001, 8832704, 8834050, 8834051, 8834054, 8834059*), O901 (*8837530*), O902 (*8834057*), R040 (*7847002, 7847003, 7847004, 7847006, 8839467*), R041 (*7848002, 7848003, 7848005, 7848006, 8830641*), R042 (*7863003, 7864002*), R048 (*4462004, 4789006, 5191010, 7863001, 8838831*), R049 (*8832240*), R18 (*7895003*), R195 (*5789012*), R233 (*2879002, 7827001, 7827002, 7827005, 7827006, 7827007, 7827008, 7827013, 8838196*), R31 (*5997003, 5997009, 5997015, 5997021, 6260001, 6266004, 6269004, 8830391, 8833170, 8835589*), R571 (*9584004*), R58 (*4590002, 4590003, 4590005, 4590006, 8832405, 8832664, 8834774, 8835227, 8837085*), S000 (*9209049, 9209053, 9209056, 9209059, 9209061, 9209062*), S001 (*8831989, 8831990, 8832021, 8832022, 8832067, 8832068, 8836485, 9209042, 9211007*), S002 (*9209066*), S003 (*8839530, 8839531*), S004 (*8831809, 8831810, 8835173, 8835174*), S005 (*8833410, 8833487, 8833488, 9209063*), S007 (*8832115, 8832116, 8838135*), S008 (*8831136, 8831865, 8832124, 8832622, 8835402*), S013 (*8727001*), S019 (*8520011*), S050 (*8831168*), S051 (*3792001, 8831765*), S063 (*8832666*), S064 (*4320005, 4320006, 4320008, 8520005, 8520016*), S0640 (*8843290, 8843322, 8843331, 8843348, 8843421, 8843544, 8843551, 8843554, 8843559, 8843576*), S0641 (*8843166, 8843173, 8843176, 8843181, 8843198, 8843289, 8843321, 8843330, 8843347, 8843420*), S065 (*4321013, 8520013, 8520015), S0650 (8842898, 8843288, 8843329, 8843496, 8843543, 8843553, 8843597*), S0651 (*8843165, 8843175, 8843217, 8843248, 8843287, 8843328, 8843495*), S066 (*8520001*), S0660 (*8843243, 8843523*), S0661 (*8843146, 8843242*), S068 (*4310040, 4321014, 8530006, 8831754, 8831759, 8831775*), S0680 (*8843251, 8843266, 8843272, 8843527, 8843534, 8843537, 8843604, 8843611*), S0681 (*8843150, 8843157, 8843160, 8843224, 8843250, 8843265, 8843271, 8843610*), S098 (*8727002*), S100 (*8830669, 8833632*), S101 (*9590035*), S141 (*8847092, 8847746*), S241 (*8847086*), S271 (*8602001*), S2710 (*8843247, 8843525*), S2711 (*8843148, 8843246*), S272 (*8831736*), S2720 (*8843245, 8843524*), S2721 (*8843147, 8843244*), S278 (*5193003*), S2780 (*8843364, 8843566*), S2781 (*8843188, 8843363*), S279 (*8832484, 8844860*), S2790 (*8843296*), S2791 (*8843295*), S301 (*8839750*), S302 (*8830687, 8830824, 8831725*), S341 (*8847142*), S361 (*8638016, 8638017*), S3610 (*8843232, 8843234, 8843519, 8843520*), S3611 (*8843142, 8843143, 8843231, 8843233*), S3680 (*8844849*), S369 (*8834673*), S3690 (*8843351, 8843560*), S3691 (*8843182, 8843350*), S370 (*8831757*), S3700 (*8843258, 8843530, 8843669*), S3701 (*8843153, 8843257*), S3780 (*8831726*), S390 (*8846320*), S400 (*8845332*), S408 (*8834009*), S500 (*8839271*), S501 (*8845361*), S600 (*8834612*), S601 (*8831532*, *8834315, 8834732, 8837572, 8840180, 9233015*), S701 (*8837353*), S800 (*8834429, 8834444, 9241017*), S801 (*8845229*), S901 (*8842493*), S902 (*8834376, 8840181*), T009 (*8837018, 8837065*), T060 (*8835980*), T090 (*8845469*), T093 (*3361012, 8835985*), T140 (*8837443, 8837446, 9249001, 9249002, 9249011, 9249014,*

**Diseases or comorbid conditions**	**ICD-10 code and/or standard disease code**
	9249027), T144 (*8831760*), T145 (*8831771*), T146 (*9249009*), T794 (*8844395*), T810 (*5258013, 8833916, 8837515, 8842779, 8844476, 8846238, 9983022*), T811 (*8844477, 9980002*), T876 (*8836052*), T905 (*8833689*), others (*8838005, 9209074*)
Ischemic stroke	I63 (*3489032*, *4371003*, *4379014*, *3448002*, *3448028*, *3489029*, *3489035*, and *4379006*)
Hemorrhagic stroke	I60–I62
Systemic embolism	I740–I745, I748, and I749

*ICD-10 codes. [†]Local standard disease codes (in parentheses).

ICD-10, 10th Revision of the International Classification of Diseases.

Table S2. Definition of diseases or comorbid conditions by ICD-10 codes and/or standard disease codes

**Diseases or comorbid conditions**	**ICD-10 code and/or standard disease codes**
Atrial fibrillation	I48
Postoperative atrial fibrillation	8847772
Valvular atrial fibrillation	8846941
Rheumatic atrial fibrillation	I05, I06, I07, I08, I09
Mechanical-valvular atrial fibrillation	T820
Hyperthyroidism or thyrotoxicosis	E05
Heart failure	I110, I500, I501 *(excluded cardiac asthma)*, I509
Hypertension	H350 *(include only hypertensive retinopathy and hypertensive neuroretinopathy),* I10, I110, I119, I120, I129 *(include only hypertensive renal disease, hypertensive nephropathy, and hypertensive nephrosclerosis)*, I150
Diabetes	E10, E11, E12, E13, E14
Hemorrhagic stroke	I60, I61, I62 *(exclude non-traumatic extradural hemorrhage)*
Ischemic stroke	I63, 3489032, 4371003, 4379014, 3448002, 3448028, 3489029, 3489035, 4379006
TIA	H340, G450, G451, G458, G459
Systemic embolism	I740 *(only abdominal aortic embolism),* I741 *(only aortic embolism),* I742 *(only acute arterial occlusive disease of the arteries of the upper extremities),* I743 *(only femoral arterial occlusion and acute arterial occlusive disease of the arteries of the lower extremities),* I744, I754 *(only iliac artery embolism),* I748 *(only hepatic artery embolism),* I749 *(only thromboembolism, embolic infarction, aortic embolism)*
Peripheral vascular disorder	I702 *(only atherosclerosis and arteriosclerosis obliterans),* I709, I731 *(only Buerger's disease),* I739 *(exclude peripheral circulatory failure, cerebrovascular spasm, and angiospasm of the extremities)*, I742, I743, I745, I748 *(only subclavian artery stenosis)*
Aortic plaque	4400011, 8837393
Coronary artery disease	I200, I201, I208, i209, I210, I211, I212, I213, I214, I240, I241, I248, I251, I252 *(exclude calcification of the coronary artery),* I255, I258 *(exclude coronary arteritis),* I259
Myocardial infarction	I200 *(exclude intermediate angina syndrome, preinfarction syndrome, intermediate coronary syndrome),* I210, I211, I212, I214, I240, I241 *(exclude Dressler syndrome)*

**Diseases or comorbid conditions**	**ICD-10 code and/or standard disease codes**
Renal dysfunction	I120, I129, I139, N003, N009, N032, N033, N039, N040, N044, N049, N052, N055, N058, N059, N170, N178, N179, N189, N19 *(exclude renal anemia, afunctional kidney, and alimentary proteinuria)*
Liver dysfunction	B150, B159, B162, B169, B171, B172, B178, B181, B182, B189, B199, K700, K701, K703, K709, K716, K720, K721, K729, K730, K732, K738, K739, K740, K741, K743, K744, K745, K746, K750, K751, K754, K759, K760, K761, K762, K763, K766, K767, K768, K769

ICD-10, 10th Revision of the International Classification of Diseases; TIA, transient ischemic attack.

Table S3. Demographics and Clinical Characteristics of Patients with 365-Day Baseline: Before s-IPTW

**Variables**	**Warfarin (N=15,902)**	**Apixaban 5/2.5 mg BID (N=22,336)**		**Dabigatran 150/110 mg BID (N=6,925)**		**Edoxaban 60/30 mg QD (N=12,262)**		**Rivaroxaban 15/10 mg QD (N=16,564)**	
	**N (%)**	**N (%)**	**Std. diff.***	**N (%)**	**Std. diff.***	**N (%)**	**Std. diff.***	**N (%)**	**Std. diff.***
Sex category (male)	9,645 (60.7)	13,205 (59.1)	-0.0313	4,741 (68.5)	0.1638	6,931 (56.5)	-0.0839	10,746 (64.9)	0.0874
Age, years									
Mean±SD	78.4±9.7	77.0±10.1	-0.1401	72.5±10.3	-0.5881	76.3±10.4	-0.2057	74.2±10.8	-0.4072
<65	1,312 (8.3)	2,298 (10.3)	0.0703	1,331 (19.2)	0.3228	1,451 (11.8)	0.1194	2,705 (16.3)	0.2479
65–74	3,405 (21.4)	5,662 (25.4)	0.0931	2,361 (34.1)	0.2861	3,181 (25.9)	0.1067	5,018 (30.3)	0.2039
≥75	11,185 (70.3)	14,376 (64.4)	-0.1277	3,233 (46.7)	-0.4945	7,630 (62.2)	-0.1722	8,841 (53.4)	-0.3547
BMI, kg/m^2									
<18	1,126 (7.1)	1,062 (4.8)	-0.0987	185 (2.7)	-0.2058	588 (4.8)	-0.0968	667 (4.0)	-0.1336
18 to <22	3,714 (23.4)	3,548 (15.9)	-0.1890	816 (11.8)	-0.3077	1,756 (14.3)	-0.2326	2,152 (13.0)	-0.2712
22 to <25	3,257 (20.5)	3,079 (13.8)	-0.1784	902 (13.0)	-0.2007	1,461 (11.9)	-0.2341	2,070 (12.5)	-0.2164
≥25	2,830 (17.8)	2,572 (11.5)	-0.1783	814 (11.8)	-0.1709	1,244 (10.2)	-0.2221	1,978 (11.9)	-0.1651
Missing	4,975 (31.3)	12,075 (54.1)	0.4732	4,208 (60.8)	0.6192	7,213 (58.8)	0.5759	9,697 (58.5)	0.5698
Dose group[†]									
Reduced	-	12,809 (57.4)	NA	5,275 (76.2)	NA	9,245 (75.4)	NA	7,812 (47.2)	NA
Standard	-	9,527 (42.7)	NA	1,650 (23.8)	NA	3,017 (24.6)	NA	8,752 (52.8)	NA
$CHADS_2$									
Mean±SD	2.5±1.5	2.3±1.5	-0.1027	2.0±1.5	-0.3471	2.2±1.5	-0.1685	2.1±1.5	-0.2503
0	1,134 (7.1)	2,597 (11.6)	0.1547	1,247 (18.0)	0.3326	1,612 (13.2)	0.2003	2,465 (14.9)	0.2496
1	3,364 (21.2)	5,178 (23.2)	0.0488	1,733 (25.0)	0.0919	2,942 (24.0)	0.0679	4,073 (24.6)	0.0819
2	3,881 (24.4)	4,659 (20.9)	-0.0848	1,559 (22.5)	-0.0447	2,606 (21.3)	-0.0752	3,700 (22.3)	-0.0489
≥3	7,523 (47.3)	9,902 (44.3)	-0.0598	2,386 (34.5)	-0.2637	5,102 (41.6)	-0.1149	6,326 (38.2)	-0.1851
CHA_2DS_2-VASc									
Mean±SD	4.1±1.8	3.9±1.9	-0.0901	3.4±1.9	-0.3891	3.8±1.9	-0.1409	3.6±1.9	-0.2748
0	260 (1.6)	612 (2.7)	0.0756	379 (5.5)	0.2084	400 (3.3)	0.1054	693 (4.2)	0.1521
1	822 (5.2)	1,529 (6.9)	0.0706	818 (11.8)	0.2400	957 (7.8)	0.1072	1,575 (9.5)	0.1670

Variables	Warfarin (N=15,902)	Apixaban 5/2.5 mg BID (N=22,336)		Dabigatran 150/110 mg BID (N=6,925)		Edoxaban 60/30 mg QD (N=12,262)		Rivaroxaban 15/10 mg QD (N=16,564)	
	**N (%)**	**N (%)**	**Std. diff.***	**N (%)**	**Std. diff.***	**N (%)**	**Std. diff.***	**N (%)**	**Std. diff.***
2	2,060 (13.0)	3,411 (15.3)	0.0666	1,231 (17.8)	0.1340	1,925 (15.7)	0.0784	2,855 (17.2)	0.1198
≥3	12,760 (80.2)	16,784 (75.1)	-0.1227	4,497 (64.9)	-0.3482	8,980 (73.2)	-0.1664	11,441 (69.1)	-0.2589
PT-INR (mean±SD)	1.6±0.7	-	-	-	-	-	-	-	-
Heart failure	7,310 (46.0)	8,049 (36.0)	-0.2030	2,049 (29.6)	-0.3428	4,442 (36.2)	-0.1990	5,564 (33.6)	-0.2549
Coronary heart disease	4,181 (26.3)	5,776 (25.9)	-0.0099	1,683 (24.3)	-0.0458	3,075 (25.1)	-0.0278	4,173 (25.2)	-0.0251
Peripheral arterial disorder	1,256 (7.9)	1,740 (7.8)	-0.0040	507 (7.3)	-0.0218	887 (7.2)	-0.0251	1,169 (7.1)	-0.0320
Myocardial infarction	506 (3.2)	676 (3.0)	-0.0090	167 (2.4)	-0.0467	362 (3.0)	-0.0133	480 (2.9)	-0.0166
Hyperthyroidism or thyrotoxicosis	289 (1.8)	450 (2.0)	0.0144	185 (2.7)	0.0577	239 (1.9)	0.0097	379 (2.3)	0.0332
Stroke, TIA, or SE	3,288 (20.7)	5,138 (23.0)	0.0563	1,335 (19.3)	-0.0350	2,452 (20.0)	-0.0169	3,247 (19.6)	-0.0268
Renal dysfunction	2,091 (13.2)	1,412 (6.3)	-0.2319	190 (2.7)	-0.3921	645 (5.3)	-0.2755	687 (4.2)	-0.3244
Liver dysfunction	1,977 (12.4)	2,758 (12.4)	-0.0026	936 (13.5)	0.0322	1,605 (13.1)	0.0197	2,028 (12.2)	-0.0057
Bleeding diagnosis	2,470 (15.5)	2,585 (11.6)	-0.1159	778 (11.2)	-0.1265	1,351 (11.0)	-0.1334	1,805 (10.9)	-0.1372
Hypertension	9,203 (57.9)	12,388 (55.5)	-0.0487	3,606 (52.1)	-0.1168	6,615 (54.0)	-0.0791	8,984 (54.2)	-0.0733
Diabetes mellitus	4,989 (31.4)	6,656 (29.8)	-0.0342	2,032 (29.3)	-0.0442	3,618 (29.5)	-0.0406	4,935 (29.8)	-0.0343
Cancer	3,884 (24.4)	5,081 (22.8)	-0.0395	1,508 (21.8)	-0.0629	2,797 (22.8)	-0.0380	3,583 (21.6)	-0.0664
Treated with antiplatelet drug	3,113 (19.6)	5,549 (24.8)	0.1270	1,562 (22.6)	0.0731	2,875 (23.5)	0.0943	3,791 (22.9)	0.0810
Treated with NSAIDs	5,771 (36.3)	6,785 (30.4)	-0.1257	1,871 (27.0)	-0.2004	3,725 (30.4)	-0.1257	4,641 (28.0)	-0.1778
Treated with gastric secretion inhibitor	6,679 (42.0)	9,246 (41.4)	-0.0123	2,510 (36.3)	-0.1181	4,834 (39.4)	-0.0525	6,378 (38.5)	-0.0713
Treated with statin-	1,733 (10.9)	3,472 (15.5)	0.1375	949 (13.7)	0.0855	1,852 (15.1)	0.1253	2,412 (14.6)	0.1101

Variables	Warfarin (N=15,902)	Apixaban 5/2.5 mg BID (N=22,336)		Dabigatran 150/110 mg BID (N=6,925)		Edoxaban 60/30 mg QD (N=12,262)		Rivaroxaban 15/10 mg QD (N=16,564)	
	N (%)	N (%)	Std. diff.*	N (%)	Std. diff.*	N (%)	Std. diff.*	N (%)	Std. diff.*
based drug									
Treated with antiarrhythmics	8,013 (50.4)	9,504 (42.6)	-0.1577	2,907 (42.0)	-0.1693	5,028 (41.0)	-0.1892	6,960 (42.0)	-0.1685
Treated with beta-blockers	3,146 (19.8)	4,634 (20.8)	0.0240	1,316 (19.0)	-0.0197	2,686 (21.9)	0.0522	3,306 (20.0)	0.0044
Treated with heparin	4,699 (29.6)	4,255 (19.1)	-0.2467	1,029 (14.9)	-0.3591	2,115 (17.3)	-0.2937	2,724 (16.5)	-0.3152
Cardioversion	139 (0.9)	138 (0.6)	-0.0298	44 (0.6)	-0.0276	72 (0.6)	-0.0337	133 (0.8)	-0.0078
Therapy days (mean±SD)	403.2±536.0	394.9±408.0	-0.0174	867.9±725.0	0.7289	264.7±263.8	-0.3277	435.7±468.4	0.0645

*Calculated when compared with the warfarin cohort. †Dose: Standard: apixaban, 5 mg BID; dabigatran, 150 mg BID; edoxaban, 60 mg QD; rivaroxaban, 15 mg QD. Reduced: apixaban, 2.5 mg BID; dabigatran, 110 mg BID; edoxaban, 30 mg QD; rivaroxaban, 10 mg QD.
BID, twice daily; BMI, body mass index; NSAID, nonsteroidal anti-inflammatory drug; PT-INR, prothrombin time–international normalized ratio; QD, once daily; SD, standard deviation; SE, systemic embolism; s-IPTW, inverse probability of treatment weighting with stabilized weights; TIA, transient ischemic attack.

Table S4. Event Rates of Major Bleeding, Any Bleeding, and Stroke/SE in Each Crude (Before s-IPTW) OAC Cohort

	**Warfarin (N=15,902)**	**Apixaban 5/2.5 mg BID (N=22,336)**	**Dabigatran 150/110 mg BID (N=6,925)**	**Rivaroxaban 15/10 mg QD (N=16,564)**	**Edoxaban 60/30 mg QD (N=12,262)**
Major bleeding					
Person-years	12,245	19,631	9,235	15,042	8,341
Number of events	341	350	125	248	149
Event rate (per 100 person-years)	2.78	1.78	1.35	1.65	1.79
Any bleeding					
Person-years	10,790	17,627	8,255	13,526	7,528
Number of events	2,134	2,736	1,066	2,007	1,434
Event rate (per 100 person-years)	19.8	15.5	12.9	14.8	19.1
Stroke/SE					
Person-years	12,296	19,682	9,221	15,096	8,372
Number of events	331	336	150	237	156
Event rate (per 100 person-years)	2.69	1.71	1.63	1.57	1.86

BID, twice daily; OAC, oral anticoagulant; QD, once daily; SE, systemic embolism; s-IPTW, inverse probability of treatment weighting with stabilized weights.

Table S5. Results of Subgroup Analysis

		Stats	Apixaban over warfarin	Dabigatran over warfarin	Edoxaban over warfarin	Rivaroxaban over warfarin
Age						
<75 years	N (NOAC/warfarin)		8,065/5,973	3,888/5,973	4,784/5,973	7,925/5,973
	Major bleeding	HR	0.74	0.58	0.85	0.75
		95% CI	[0.535–1.012]	[0.396–0.852]	[0.578–1.247]	[0.540–1.046]
		p-value	0.0589	0.0054	0.4042	0.0907
	Any bleeding	HR	0.97	0.88	1.05	0.95
		95% CI	[0.871–1.075]	[0.776–0.997]	[0.926–1.183]	[0.857–1.064]
		p-value	0.5431	0.0448	0.4679	0.4005
	Stroke/SE	HR	0.73	0.83	0.80	0.61
		95% CI	[0.527–1.000]	[0.577–1.186]	[0.546–1.186]	[0.437–0.866]
		p-value	0.0501	0.3025	0.2718	0.0053
≥75 years	N (NOAC/warfarin)		14,605/12,702	3,766/12,702	7,835/12,702	9,161/12,702
	Major bleeding	HR	0.71	0.72	0.66	0.73
		95% CI	[0.590–0.843]	[0.544–0.951]	[0.522–0.836]	[0.595–0.898]
		p-value	0.0001	0.0207	0.0006	0.0028
	Any bleeding	HR	0.90	1.00	1.02	0.93
		95% CI	[0.836–0.966]	[0.893–1.111]	[0.934–1.107]	[0.856–1.008]
		p-value	0.0038	0.9397	0.7049	0.0756
	Stroke/SE	HR	0.61	0.78	0.68	0.72
		95% CI	[0.512–0.736]	[0.599–1.024]	[0.540–0.857]	[0.591–0.887]
		p-value	<0.0001	0.0740	0.0011	0.0019
<80 years	N (NOAC/warfarin)		12,438/9,595	5,431/9,595	7,331/9,595	11,300/9,595
	Major bleeding	HR	0.76	0.62	0.84	0.81
		95% CI	[0.601–0.964]	[0.463–0.833]	[0.628–1.119]	[0.637–1.038]
		p-value	0.0234	0.0015	0.2318	0.0968
	Any bleeding	HR	0.95	0.91	1.05	0.96
		95% CI	[0.879–1.035]	[0.828–1.010]	[0.955–1.153]	[0.886–1.050]
		p-value	0.2577	0.0785	0.3188	0.3997

		Stats	Apixaban over warfarin	Dabigatran over warfarin	Edoxaban over warfarin	Rivaroxaban over warfarin
	Stroke/SE	HR	0.68	0.78	0.73	0.66
		95% CI	[0.540–0.866]	[0.593–1.024]	[0.545–0.974]	[0.517–0.855]
		p-value	0.0016	0.0736	0.0327	0.0014
≥80 years	N (NOAC/warfarin)		10,234/9,103	2,131/9,103	5,305/9,103	5,806/9,103
	Major bleeding	HR	0.67	0.74	0.60	0.65
		95% CI	[0.546–0.829]	[0.522–1.041]	[0.452–0.797]	[0.506–0.842]
		p-value	0.0002	0.0831	0.0004	0.0010
	Any bleeding	HR	0.88	1.02	0.99	0.90
		95% CI	[0.805–0.959]	[0.887–1.179]	[0.894–1.102]	[0.813–0.996]
		p-value	0.0037	0.7572	0.8893	0.0422
	Stroke/SE	HR	0.62	0.82	0.70	0.74
		95% CI	[0.500–0.765]	[0.589–1.146]	[0.537–0.922]	[0.582–0.947]
		p-value	<0.0001	0.2478	0.0108	0.0166
Body weight						
<60 kg	N (NOAC/warfarin)		6,551/7,274	1,662/7,274	3,493/7,274	3,986/7,274
	Major bleeding	HR	0.86	0.99	0.76	0.99
		95% CI	[0.659–1.115]	[0.658–1.475]	[0.534–1.091]	[0.732–1.341]
		p-value	0.2515	0.9417	0.1379	0.9534
	Any bleeding	HR	0.99	1.05	1.01	0.95
		95% CI	[0.889–1.094]	[0.887–1.252]	[0.887–1.149]	[0.838–1.071]
		p-value	0.7953	0.5489	0.8871	0.3845
	Stroke/SE	HR	0.75	0.95	0.93	1.01
		95% CI	[0.580–0.973]	[0.645–1.389]	[0.674–1.279]	[0.756–1.351]
		p-value	0.0302	0.7796	0.6500	0.9428
≥60 kg	N (NOAC/warfarin)		3,993/4,631	1,486/4,631	1,794/4,631	3,190/4,631
	Major bleeding	HR	0.60	0.47	0.89	0.47
		95% CI	[0.429–0.853]	[0.296–0.742]	[0.574–1.379]	[0.313–0.717]
		p-value	0.0041	0.0012	0.6008	0.0004
	Any bleeding	HR	0.86	0.98	0.98	0.83

		Stats	Apixaban over warfarin	Dabigatran over warfarin	Edoxaban over warfarin	Rivaroxaban over warfarin
		95% CI	[0.759–0.975]	[0.830–1.149]	[0.832–1.153]	[0.721–0.951]
		p-value	0.0185	0.7762	0.8061	0.0077
	Stroke/SE	HR	0.72	0.84	0.80	0.73
		95% CI	[0.507–1.021]	[0.549–1.295]	[0.501–1.263]	[0.496–1.070]
		p-value	0.0656	0.4357	0.3328	0.1059
Renal disease						
NO	N (NOAC/warfarin)		21,284/16,011	7,568/16,011	11,877/16,011	16,544/16,011
	Major bleeding	HR	0.78	0.80	0.79	0.72
		95% CI	[0.629–0.977]	[0.587–1.091]	[0.588–1.054]	[0.561–0.936]
		p-value	0.0304	0.1581	0.1082	0.0136
	Any bleeding	HR	0.95	1.02	0.98	0.91
		95% CI	[0.875–1.038]	[0.913–1.150]	[0.878–1.088]	[0.825–1.001]
		p-value	0.2710	0.6832	0.6772	0.0524
	Stroke/SE	HR	0.84	0.88	0.97	0.83
		95% CI	[0.680–1.050]	[0.654–1.172]	[0.738–1.282]	[0.651–1.066]
		p-value	0.1277	0.3716	0.8447	0.1459
YES	N (NOAC/warfarin)		1,471/2,201	247/2,201	694/2,201	754/2,201
	Major bleeding	HR	0.75	0.59	1.48	1.81
		95% CI	[0.371–1.504]	[0.081–4.293]	[0.633–3.445]	[0.851–3.856]
		p-value	0.4144	0.6024	0.3677	0.1231
	Any bleeding	HR	0.88	1.03	1.30	0.88
		95% CI	[0.687–1.125]	[0.588–1.792]	[0.951–1.770]	[0.625–1.246]
		p-value	0.3056	0.9254	0.1002	0.4779
	Stroke/SE	HR	0.31	2.47	1.38	2.85
		95% CI	[0.107–0.917]	[0.946–6.440]	[0.576–3.309]	[1.440–5.650]
		p-value	0.0342	0.0649	0.4693	0.0026
Concomitant use of antiplatelet medication						
NO	N (NOAC/warfarin)		17,072/15,145	6,146/15,145	9,646/15,145	13,439/15,145
	Major bleeding	HR	0.84	1.63	1.67	1.11

		Stats	Apixaban over warfarin	Dabigatran over warfarin	Edoxaban over warfarin	Rivaroxaban over warfarin
		95% CI	[0.371–1.895]	[0.666–3.971]	[0.704–3.941]	[0.450–2.732]
		p-value	0.6710	0.2851	0.2453	0.8217
	Any bleeding	HR	0.94	1.33	1.33	0.97
		95% CI	[0.688–1.272]	[0.855–2.072]	[0.928–1.908]	[0.699–1.347]
		p-value	0.6719	0.2058	0.1202	0.8573
	Stroke/SE	HR	0.53	1.34	0.45	0.42
		95% CI	[0.231–1.232]	[0.494–3.612]	[0.130–1.587]	[0.155–1.133]
		p-value	0.1411	0.5690	0.2165	0.0867
YES	N (NOAC/warfarin)		5,683/3,915	1,889/3,915	2,979/3,915	4,065/3,915
	Major bleeding	HR	0.25	0.00	0.93	0.14
		95% CI	[0.065–0.952]	[0.000–0.000]	[0.250–3.441]	[0.017–1.140]
		p-value	0.0421	<0.0001	0.9111	0.0662
	Any bleeding	HR	0.83	0.48	1.27	0.60
		95% CI	[0.519–1.317]	[0.200–1.150]	[0.699–2.305]	[0.329–1.104]
		p-value	0.4222	0.0996	0.4342	0.1011
	Stroke/SE	HR	0.37	0.18	1.47	0.87
		95% CI	[0.087–1.578]	[0.020–1.611]	[0.393–5.483]	[0.195–3.895]
		p-value	0.1791	0.1254	0.5674	0.8562
Dosage						
Standard	N (NOAC/warfarin)		10,380/27,915	2,781/27,915	3,986/27,915	10,287/27,915
	Major bleeding	HR	0.31	0.28	2.10	1.53
		95% CI	[0.072–1.352]	[0.037–2.133]	[0.588–7.475]	[0.366–6.401]
		p-value	0.1194	0.2196	0.2541	0.5606
	Any bleeding	HR	0.76	0.55	2.05	1.08
		95% CI	[0.485–1.208]	[0.235–1.282]	[1.137–3.695]	[0.679–1.717]
		p-value	0.2501	0.1656	0.0170	0.7468
	Stroke/SE	HR	0.41	0.90	1.57	0.18
		95% CI	[0.119–1.425]	[0.244–3.300]	[0.361–6.819]	[0.024–1.329]
		p-value	0.1611	0.8705	0.5481	0.0925

		Stats	Apixaban over warfarin	Dabigatran over warfarin	Edoxaban over warfarin	Rivaroxaban over warfarin
Reduced	N (NOAC/warfarin)		14,408/17,712	6,858/17,712	9,763/17,712	8,093/17,712
	Major bleeding	HR	0.62	1.05	1.08	0.85
		95% CI	[0.281–1.371]	[0.401–2.732]	[0.475–2.444]	[0.347–2.061]
		p-value	0.2378	0.9255	0.8588	0.7118
	Any bleeding	HR	0.95	1.23	1.15	0.86
		95% CI	[0.704–1.286]	[0.759–1.987]	[0.813–1.624]	[0.602–1.218]
		p-value	0.7449	0.4019	0.4296	0.3890
	Stroke/SE	HR	0.40	1.07	0.63	0.72
		95% CI	[0.177–0.914]	[0.357–3.201]	[0.233–1.690]	[0.304–1.710]
		p-value	0.0297	0.9048	0.3566	0.4573

CI, confidence interval; HR, hazard ratio; NOAC, non–vitamin K oral anticoagulant; SE, systemic embolism.

Table S6. Results of sensitivity analysis: observation period

	Stats	Apixaban over warfarin	Dabigatran over warfarin	Edoxaban over warfarin	Rivaroxaban over warfarin
	N (NOAC/warfarin)	22,752/19,059	8,003/19,059	12,592/19,059	17,481/19,059
Observational period: 1 year					
Major bleeding	HR	0.66	0.61	0.69	0.77
	95% CI	[0.551–0.799]	[0.459–0.803]	[0.554–0.865]	[0.631–0.941]
	p-value	<0.0001	0.0005	0.0012	0.0106
Any bleeding	HR	0.95	0.96	1.05	0.96
	95% CI	[0.891–1.014]	[0.871–1.049]	[0.971–1.127]	[0.894–1.029]
	p-value	0.1225	0.3377	0.2369	0.2492
Stroke/SE	HR	0.68	0.86	0.68	0.71
	95% CI	[0.565–0.811]	[0.676–1.093]	[0.548–0.853]	[0.575–0.866]
	p-value	<0.0001	0.2171	0.0008	0.0008
Observational period: 2 years					
Major bleeding	HR	0.72	0.66	0.71	0.74
	95% CI	[0.614–0.843]	[0.529–0.825]	[0.583–0.874]	[0.618–0.879]
	p-value	<0.0001	0.0003	0.0011	0.0007
Any bleeding	HR	0.93	0.96	1.03	0.94
	95% CI	[0.872–0.984]	[0.881–1.039]	[0.958–1.103]	[0.881–1.004]
	p-value	0.0127	0.2893	0.4404	0.0640
Stroke/SE	HR	0.65	0.79	0.72	0.71
	95% CI	[0.558–0.766]	[0.642–0.977]	[0.591–0.879]	[0.592–0.842]
	p-value	<0.0001	0.0291	0.0012	0.0001

The 2-year and 1-year observational period was the result of the main analysis and sensitivity analysis, respectively.
CI, confidence interval; HR, hazard ratio; NOAC, non–vitamin K oral anticoagulant; SE, systemic embolism.

Table S7. Results of sensitivity analysis: methods to balance patient characteristics

	Stats	Apixaban over warfarin	Dabigatran over warfarin	Edoxaban over warfarin	Rivaroxaban over warfarin
	N (NOAC/warfarin)	14,428/14,428	6,402/6,402	10,808/10,808	11,930/11,930
Propensity score matching					
Major bleeding	HR	0.73	0.65	0.69	0.73
	95% CI	[0.620–0.867]	[0.505–0.828]	[0.555–0.854]	[0.607–0.881]
	p-value	0.0003	0.0005	0.0007	0.0010
Any bleeding	HR	0.95	0.93	1.01	0.97
	95% CI	[0.895–1.016]	[0.850–1.023]	[0.934–1.088]	[0.907–1.043]
	p-value	0.1396	0.1373	0.8364	0.4372
Stroke/SE	HR	0.65	0.74	0.67	0.75
	95% CI	[0.546–0.777]	[0.585–0.934]	[0.542–0.833]	[0.619–0.902]
	p-value	<0.0001	0.0113	0.0003	0.0024
s-IPTW					
Major bleeding	HR	0.72	0.66	0.71	0.74
	95% CI	[0.614–0.843]	[0.529–0.825]	[0.583–0.874]	[0.618–0.879]
	p-value	<0.0001	0.0003	0.0011	0.0007
Any bleeding	HR	0.93	0.96	1.03	0.94
	95% CI	[0.872–0.984]	[0.881–1.039]	[0.958–1.103]	[0.881–1.004]
	p-value	0.0127	0.2893	0.4404	0.0640
Stroke/SE	HR	0.65	0.79	0.72	0.71
	95% CI	[0.558–0.766]	[0.642–0.977]	[0.591–0.879]	[0.592–0.842]
	p-value	<0.0001	0.0291	0.0012	0.0001

The observational period was 2 years after the index date for both analyses.

CI, confidence interval; HR, hazard ratio; NOAC, non–vitamin K oral anticoagulant; SE, systemic embolism; s-IPTW, inverse probability of treatment weighting with stabilized weights.
